# Integrating Abiotic Stress Adaptation and Disease Resistance in Climate-Resilient Crops: Pangenomics, Genomic Selection, and AI-Driven Breeding

**DOI:** 10.3390/plants15203171

**Published:** 2026-10-16

**Authors:** Yousol Hwang, Gwangmin Ryu, Moon-Sub Lee, Yoon-Sup So, Ju-Kyung Yu, Bong-Gyu Mun

**Affiliations:** 1 Department of Crop Science, Chungbuk National University, Cheongju 28644, Republic of Korea; 2 Department of Environmental and Biological Chemistry, Chungbuk National University, Cheongju 28644, Republic of Korea

**Keywords:** genomic selection, pangenome, quantitative disease resistance, multi-omics, artificial intelligence, climate change, crop disease resistance breeding

## Abstract

Climate change is increasing the frequency and intensity of abiotic stresses, including drought, high temperature, salinity, and waterlogging, which not only directly constrain crop growth and productivity but also reshape plant–pathogen interactions by altering host physiology and immune responses. These climate-driven environmental pressures can accelerate pathogen spread and generational turnover while compromising crop disease resistance through complex interactions between abiotic stress responses and plant immunity. Concurrently, modern cultivars have lost a substantial portion of their genetic diversity through prolonged productivity-focused selection, limiting their capacity to respond to rapidly changing environmental and disease pressures. Consequently, resistance strategies relying primarily on a few major resistance genes and Marker-Assisted Selection (MAS) are becoming increasingly insufficient for maintaining stable crop performance under climate change. This review discusses next-generation strategies for developing climate-resilient disease-resistant crops through three complementary approaches. First, we examine quantitative disease resistance (QDR), which is typically governed by multiple loci with small to moderate effects and can contribute to durable resistance, although its effectiveness depends on the host genotype, pathogen isolate, and environment. We also evaluate the application of genomic selection (GS) to predict quantitative resistance and select individuals with favorable genomic estimated breeding values across major crops. Third, we highlight AI-driven precision breeding that integrates genomic, transcriptomic, metabolomic, phenomic, and environmental information to decipher complex genotype-by-environment (G × E) interactions and improve the prediction of stress-associated disease resistance. Collectively, integrating genetic diversity, genome-wide prediction, multi-omics, and AI provides a framework for breeding toward durable disease resistance and stable productivity under increasingly complex abiotic stress environments, with resistance performance requiring validation across relevant pathogen isolates and environments.

## 1. Introduction

As climate change intensifies globally, the survival ranges of pathogens that threaten crop production and the patterns of disease occurrence are shifting rapidly [1]. Fluctuations in temperature, precipitation, and humidity accelerate pathogen infection cycles and shorten their generational turnover, while compound environmental stresses disrupt innate plant immune systems, thereby structurally increasing crop vulnerability to diseases [2,3]. Beyond mere yield losses, this poses a severe threat to overall food security when coupled with the imperative to meet the food demands of a growing global population stably [4]. The crops that must withstand these external pressures are themselves vulnerable. Through decades of productivity-focused breeding, modern cultivars have lost a substantial portion of the genetic diversity required to counter pest and disease pressures. Pedigree analysis of 258 modern public soybean cultivars released in the United States between 1947 and 1988 revealed that just 28 ancestors and 7 first-generation progenies accounted for 95% of the total pedigree. Molecular-level analyses further demonstrated that modern cultivars retained only 72% of the sequence diversity found in Asian landraces, while losing 79% of rare alleles. The most severe bottleneck occurred during domestication, with cultivated soybean retaining only 66% of the nucleotide diversity of wild soybean (*G. soja*). Furthermore, analysis of the USDA soybean germplasm collection indicated a high level of redundancy within the collection, as 30% of *G. soja* and 23% of *G. max* accessions exhibited more than 99.9% genetic similarity [5,6,7].

In a study utilizing the NJAU 355K SoySNP array, Wang et al. [8] compared the genetic variation of 105 wild and 262 cultivated soybean accessions, revealing that the nucleotide diversity (π) of wild soybean was significantly higher than that of cultivated soybean (P = 1.92 × 10^−28^) [8]. Notably, 13,757 SNPs were identified as polymorphic in *G. soja* but monomorphic in *G. max*, suggesting that these loci were subjected to artificial selection. Furthermore, cultivated soybean exhibited lower Tajima’s D values compared to wild populations, confirming the presence of recent selective sweeps, and the linkage disequilibrium (LD) decay distance was maintained longer in cultivated soybean (130 kb) than in wild soybean (80 kb), clearly demonstrating the impact of artificial selection. Tao et al. [9] constructed an AA-genome wild rice pangenome using genome resequencing data from 180 wild and 39 cultivated rice accessions. The results indicated that between 15,730 and 26,882 genes were lost during the evolution from wild rice to cultivated rice (*O. sativa*), with more than 40% of the AA-genome wild rice genes disappearing throughout this process. The nucleotide diversity of cultivated rice was significantly lower than that of wild rice, and *O. rufipogon* exhibited the highest nucleotide diversity (0.00568), confirming the genetic bottleneck effect of domestication. Notably, Gene Ontology (GO) enrichment analysis of the presence–absence variation (PAV) genes detected in the wild rice pangenome revealed that the top 15 significant GO terms were mostly related to stress responses [9]. This suggests that genes contributing to pest and disease resistance, as well as environmental adaptation, may have been preferentially lost during domestication. Furthermore, functional analysis of genes located within selective sweep regions also identified numerous genes involved in stress responses and growth regulation. In a comprehensive review of pangenome studies across multiple crops, Petereit et al. [10] pointed out that domestication and breeding systematically reduce the frequency of dispensable genes. In soybean, the proportion of dispensable genes decreased stepwise from 10.17% in wild accessions (*G. soja*) to 9.06% in landraces (*G. max*), and further to 8.69% in modern cultivars. Similarly, in tomato, this proportion progressively declined from 20.98% in wild species to 18.60% in landraces, and to 16.11% in modern cultivars [10]. In soybean, dispensable genes are enriched for functions associated with defense, salt stress, and other biotic and abiotic stress responses [11]. In three *Brassica* pangenomes, dispensable genes are likewise enriched for defense-related functions, although the mechanisms of gene loss differ between diploid and polyploid species [12]. Loss of such variation may reduce the genetic resources available for environmental adaptation. Petereit et al. [10] discussed a reported negative association between nucleotide-binding leucine-rich repeat (NLR) resistance gene content and yield in soybean, suggesting that selection for higher yield may be accompanied by changes in resistance gene content. This association should not be interpreted as a universal or causal relationship across crops; the resistance–yield relationship depends on genetic background and growing conditions [13]. These findings support broader use of crop genetic diversity [14,15]. Major-gene resistance can break down when new virulent pathogen races emerge, while Marker-Assisted Selection (MAS) remains difficult to apply efficiently to complex resistance controlled by many minor-effect loci [16,17,18]. This paper examines the paradigm shift in breeding to overcome this complex crisis, focusing on three strategic axes. First, we review the importance of Quantitative Disease Resistance (QDR), which can provide broad-spectrum and relatively durable defense, and evaluate the applicability of Genomic Selection (GS) across major crops for the simultaneous genome-wide prediction and selection of multiple minor-effect genes. Second, we discuss the necessity of adopting pangenome approaches as critical tools to restore lost genetic diversity and uncover structural variations and resistance genes that single-reference genomes overlook. Third, to translate pangenome and GS outcomes into practical breeding applications, we propose a data-driven precision breeding strategy that integrates genomic, transcriptomic, metabolomic, phenomic, and environmental data, using artificial intelligence (AI) to precisely decipher complex genotype-by-environment (G × E) interactions.

### Literature Search and Review Approach

This article was conducted as a narrative review to integrate current knowledge on crop genetic diversity, quantitative disease resistance, pangenomics, genomic selection, and AI-assisted breeding under climate change. Relevant literature was identified through searches of Web of Science, Scopus, PubMed, and Google Scholar, with emphasis on studies mainly published from 2000 to 2026; earlier seminal studies were included where necessary to provide historical or conceptual context. Search terms included combinations of “climate change,” “crop disease resistance,” “abiotic stress,” “genetic diversity,” “genetic erosion,” “crop wild relatives,” “quantitative disease resistance” or “QDR,” “pangenome” or “pan-genome,” “dispensable genes,” “NLR,” “structural variation,” “presence–absence variation,” “genomic selection,” “genomic prediction,” “machine learning,” “deep learning,” “multi-omics,” and “genotype-by-environment interaction,” “yield,” and “growing season.” Relevant studies were additionally identified by examining the reference lists of key reviews and primary research articles.

Studies were prioritized when they provided experimental, mechanistic, or prediction-based evidence relevant to crop resistance breeding, genetic diversity, or adaptation to changing environments. Particular attention was given to major crops, including wheat, rice, maize, soybean, barley, potato, and *Brassica* crops. Studies in model species or other biological systems were included when they provided transferable mechanistic or methodological insights, but were not treated as direct evidence of validated crop-breeding outcomes. Reviews were used for conceptual background, and authoritative reports were included to provide agricultural context. Studies with limited relevance to the themes of this review were not prioritized. Selected studies were compared according to the crop–pathogen system, population or germplasm, stress or disease conditions, data inputs, prediction model, reported performance, and validation design. Cross-validation was distinguished from forward or independent validation, and statistical associations and predictions were distinguished from functional validation and demonstrated breeding outcomes. DOI and bibliographic information were checked against publisher pages and Crossref records. Because the purpose was an integrative narrative synthesis rather than a systematic assessment of intervention effects, no formal meta-analysis or standardized risk-of-bias assessment was performed. Differences in study design, environments, and performance metrics were considered when interpreting the evidence, and the potential for selection and publication biases was acknowledged.

## 2. Climate Change and the Crisis of Crop Genetic Diversity

### 2.1. Shifts in Disease Occurrence Patterns Driven by Extreme Weather

Rapid climate change extends beyond mere increases in mean temperatures, escalating the frequency and intensity of extreme weather events such as droughts, heavy rainfall, floods, and heatwaves, thereby altering crop productivity and disease patterns. Changes in temperature, precipitation, humidity, and atmospheric CO_2_ concentrations affect not only crop growth conditions but also pathogen survival, infection, dissemination, overwintering, and overall host–pathogen interactions, consequently shifting the timing, severity, and geographic distribution of disease outbreaks. Zhao et al. [19] reported that, excluding CO_2_ fertilization effects and adaptation, each 1 °C increase in global mean temperature would lead to yield reductions of 6.0% in wheat, 3.2% in rice, 7.4% in maize, and 3.1% in soybean [19]. These declines are driven not only by the direct effects of rising temperatures but also by indirect pathways, including reduced soil moisture, shorter growth periods, more frequent heatwaves, and altered pest and disease pressures. Furthermore, using a mixed-effects model that incorporates precipitation changes and temperature-precipitation interactions, Li et al. [20] projected global yield reductions by 2080–2100 under the high-emission scenario (SSP5-8.5) of −22% for maize, −9% for rice, −15% for soybean, and −14% for wheat. Concurrent high temperatures and drought can compound damage beyond the simple sum of individual stresses, leading to higher estimated losses for maize and rice than previously analyzed [20].

Climate change is also rapidly transforming the cultivation environment itself. An analysis of long-term field experiment sites in Germany projected that under the SSP5-8.5 scenario, numerous regions will transition from humid or sub-humid to semi-arid conditions, frost days will decrease substantially, and growing degree days (GDD) will increase [21]. This implies that such changes can alter crop growth durations, sowing dates, exposure to heat stress, and water availability, thereby undermining the stability of existing cropping systems. Consequently, these altered cultivation environments simultaneously shift the conditions for disease occurrence. Rather than being explained solely by increases in mean temperature, disease incidence is heavily dictated by weather anomalies. An analysis of global plant disease data revealed a non-linear pattern in wild plants, with disease prevalence peaking when temperatures are approximately 2.7 °C above the local historical average. Furthermore, anomalously high temperatures in cold regions and anomalously heavy rainfall in arid regions were associated with increased disease, suggesting that climatic maladaptation and precipitation mismatches are critical drivers of disease outbreaks. Conversely, agricultural systems exhibited vulnerability where current high temperatures themselves increase disease risks, despite anthropogenic management practices such as irrigation, pesticide application, and cultivar selection [22]. Extreme weather events also exert a direct impact on the life cycles of pathogens. Under high-temperature and high-humidity conditions, the latent periods and generation times of many fungal pathogens are shortened, and recurrent infection cycles within a growing season increase, leading to higher disease pressure over the same timeframe. This accelerates the generational turnover of pathogens, expands population sizes, and repeatedly imposes strong environmental selection pressure, thereby elevating the likelihood of the emergence and dissemination of new races. Consequently, this heightens the risk of the rapid breakdown of disease resistance in existing crops [23,24].

Climate change can also create regional opportunities for crop production. In cool, high-latitude regions, fewer frost days and a longer frost-free season may permit earlier sowing, longer-duration cultivars, or expansion into areas previously limited by low temperature [21,25]. A longer favorable season does not automatically increase yield: warming may also accelerate phenological development, so sowing dates and cultivar maturity must be adapted to use the additional growing window [25]. Elevated CO_2_ can stimulate productivity in C3 crops, although field responses are generally smaller than earlier enclosure-based estimates and depend on crop and growing conditions [26]. These potential benefits can be constrained by water or nutrient limitation and by heat, pest, and disease pressures. Breeding targets should therefore be region-specific, combining suitable flowering and maturity responses, efficient resource use, and disease resistance to exploit favorable conditions while maintaining yield stability [10,25,26].

### 2.2. Compound Environmental Stress and Compromised Plant Immunity

Extreme weather events not only directly affect the life cycles of pathogens but also deeply impact the defense mechanisms of host plants. For instance, drought stress extends beyond merely inhibiting plant growth; it directly engages in disease resistance regulatory networks mediated by ABA signaling. Notably, under combined stress conditions, drought-activated ABA signaling has been reported to suppress the CBP60g–SARD1 module, thereby compromising SA-mediated immunity and consequently increasing susceptibility to bacterial pathogens [27]. Submergence stress likewise compromises the physiological defense capabilities of host plants and disrupts the balance of the microbiome, thereby creating an environment favorable for the invasion and colonization of pathogens [28]. Thus, climate change should be viewed not as a problem of isolated stressors but as the emergence of a novel disease environment in which compound environmental stresses and pathogen pressures operate simultaneously.

### 2.3. The Dual Crisis: Production Loss and the Erosion of Genetic Diversity

Extreme weather events are exerting a substantial impact on crop yields. In fact, it is reported that between 1983 and 2009, three-quarters of global harvested areas experienced production losses due to drought, resulting in accumulated losses of approximately $166 billion [29]. According to the latest FAO report, global agricultural losses over the 33-year period from 1991 to 2023 totaled approximately $3.26 trillion. The scale of these losses has intensified recently, increasing from an annual average of about $64 billion in the 1990s to $144 billion since 2010 [30]. With the global population projected to reach 9.8 billion by 2050, such production losses pose a significant threat to food security. However, a more critical issue under climate change is that while the evolution and dissemination of pathogens are accelerating, modern cultivars have progressively lost the genetic diversity necessary to counter them. Through prolonged selection and breeding to meet human demands, modern cultivars have accumulated traits focused on productivity, uniformity, and adaptability to cultivation. In this process, the genetic diversity required to respond to novel environmental changes and pest pressures has been severely diminished. Consequently, today’s cultivars have become increasingly vulnerable to emerging disease pressures driven by climate change.

## 3. Limitations of Conventional Disease Resistance Breeding and the Necessity of Genomic Selection

### 3.1. Quantitative Resistance and the Need for GS-Based Selection Strategies

Crop disease resistance is categorized as quantitative or qualitative based on the genetic regulatory mechanisms involved. Traditional breeding has primarily targeted qualitative disease resistance, governed by one or two large-effect alleles known as resistance genes (R genes) or major genes [31,32,33,34]. Due to its high heritability and ease of phenotypic identification, which allows for early-stage selection, qualitative resistance has long served as the core strategy for developing resistant cultivars [34,35]. Advances in high-throughput SNP marker technology have accelerated the introgression of these qualitative resistance genes. Marker-Assisted Selection (MAS) has enabled the rapid incorporation of major R genes into elite cultivars, significantly enhancing breeding efficiency [17,36,37]. However, qualitative resistance based on a single major resistance gene—the primary target of MAS—exhibits a fundamental limitation: while highly effective against specific pathogen races, it can be rapidly neutralized upon the emergence of new virulent races within the pathogen population [38,39,40]. To overcome this, increasing importance is being placed on Quantitative Disease Resistance (QDR). QDR is a continuously varying reduction in disease development among host genotypes, usually governed by several quantitative resistance loci (QRLs) with small to moderate effects. It is expressed as partial resistance rather than a simple resistant/susceptible phenotype and can be measured through infection frequency, lesion size, pathogen growth or sporulation, latency period, or the area under the disease progress curve (AUDPC) [41,42,43]. Resistance loci may act at different stages of infection and need not encode canonical pathogen-recognition receptors. QDR often provides broader and more durable protection than single major-gene resistance, but race nonspecificity and environmental stability are not defining guarantees: its expression can depend on pathogen isolate, host developmental stage, and environment. Accordingly, breeding requires quantitative phenotyping across relevant isolates and environments [41,43,44]. Due to these characteristics, QDR is gaining attention as a sustainable resistance strategy that is less likely to be overcome by pathogen evolution compared to single-R-gene-based resistance, despite being a partial resistance involving multiple loci [41,45].

Recent QDR research has expanded into QTL mapping, genome-wide association studies (GWAS), fine mapping, marker development, genomic selection (GS), high-throughput phenotyping, and multi-omics-based prediction. QDR-related loci and breeding strategies have been reported in various crop–pathogen systems, including blackleg in canola, white mold in common bean, leaf spot in peanut, and rust/*Fusarium* head blight in wheat [42,43,45]. In soybean, polygenic partial resistance is also considered a critical form of resistance to diseases such as soybean cyst nematode, sudden death syndrome, and *Sclerotinia* stem rot. Improving these complex resistance traits requires prediction-based breeding strategies that integrate genome-wide marker effects rather than relying solely on individual marker-based selection [46].

Genomic Selection (GS) is a method that utilizes high-density marker information distributed across the entire genome to simultaneously predict and select for the Genomic Estimated Breeding Value (GEBV) of individuals. GS is considered a highly suitable approach for improving polygenic disease resistance traits like QDR because it comprehensively reflects the numerous minor effects contributing to a trait as genome-wide marker effects, rather than relying only on a few statistically significant markers [47,48]. Numerous studies have reported that GS-based whole-genome prediction models achieve prediction accuracy and selection efficiency suitable for QDR breeding. Poland and Rutkoski [18] synthesized GS applications across various crop–disease systems, including wheat rust, *Fusarium* head blight (FHB), northern corn leaf blight, and cassava mosaic disease. Prediction accuracies for rust resistance ranged from 0.39 to 0.85 in cross-validation and from 0.20 to 0.40 in forward validation, while accuracies of 0.47 to 0.76 were reported for predicting deoxynivalenol (DON) content associated with FHB [18]. They emphasized that since GS estimates all genome-wide marker effects simultaneously, it can capture numerous minor-effect loci often missed by MAS. Furthermore, they highlighted that if genomic information is already available for existing breeding populations, disease resistance prediction can be applied at no additional cost. In research on wheat stripe rust resistance, rrBLUP-based GS models showed prediction accuracies of up to 0.72 for disease severity, consistently outperforming MAS models.

Notably, adding major resistance gene markers as fixed effects resulted in limited improvements in accuracy, suggesting that genome-wide markers alone can effectively predict disease resistance involving both major and minor genes [49]. Furthermore, a comparison of genome-wide prediction models for *Septoria tritici* blotch, *Stagonospora nodorum* blotch, and tan spot resistance in CIMMYT wheat breeding populations revealed that GS models—such as GBLUP, Bayesian models, and RKHS—achieved prediction accuracies on average 48% higher than simple marker-based approaches [50]. GS has also been proposed as a strategy to complement or replace phenotypic selection in practical breeding stages. In a forward prediction study targeting FHB resistance in a U.S. soft red winter wheat breeding program, the GS model accurately selected approximately 69–70% of superior lines based on DON content, whereas phenotypic selection achieved only 33%. This demonstrates that GS can enhance the efficiency of disease resistance selection under conditions where disease occurrence is non-uniform or phenotypic evaluation accuracy is low [51]. Meanwhile, Michel et al. [52] suggested through simulation studies that the design of the training population is crucial for simultaneously improving R-gene-based qualitative resistance and background quantitative resistance [52]. They proposed that a strategy of isolating and predicting QDR to ensure it is not masked by R gene effects is advantageous for long-term resistance accumulation. In summary, GS-based whole-genome models have demonstrated practical prediction accuracies generally ranging from 0.4 to 0.7 across various crop–disease systems, such as wheat rust, FHB, leaf spot, and corn leaf blight.

**Figure 1:**
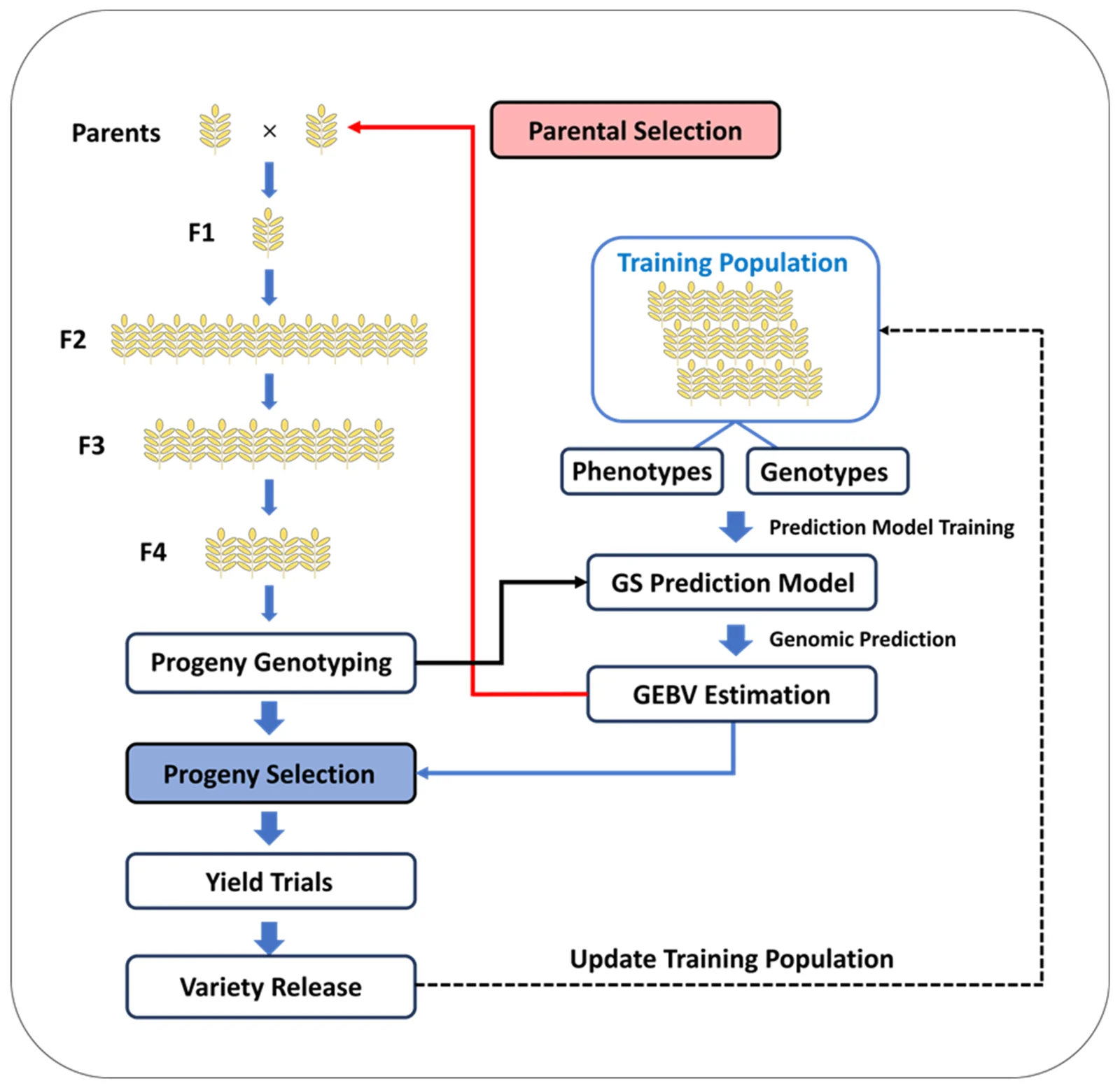
Integration of genomic selection (GS) into a conventional crop breeding pipeline. The conventional breeding process involves parental selection, crossing, generation advancement, progeny selection, yield trials, and eventual variety release. In the GS-assisted pathway, a training population with both phenotypic and genotypic information is used to develop a genomic prediction model. The resulting model estimates genomic estimated breeding values (GEBVs) for genotyped candidate progenies, enabling promising lines to be prioritized at the progeny selection stage before extensive field evaluation. Thus, GS complements rather than replaces conventional breeding by providing genome-wide prediction to support early selection decisions.

These models are evaluated as more effective than MAS—which relies on a few key markers—in predicting complex quantitative disease resistance traits. These findings support the necessity for disease resistance breeding in the era of climate change to expand beyond the short-term introduction of major genes toward GS-based strategies that simultaneously capture and accumulate numerous minor-effect resistance loci dispersed throughout the genome. In practical breeding programs, GS does not replace the conventional breeding process but is integrated into specific stages where early prediction and selection can improve breeding efficiency. Following parental selection, crossing, and generation advancement, candidate progenies can be genotyped before extensive field evaluation. A genomic prediction model developed from a training population with both phenotypic and genotypic data is then used to estimate genomic estimated breeding values (GEBVs) for these progenies. Lines with favorable GEBVs can be prioritized for subsequent phenotypic evaluation and yield trials, thereby reducing the number of individuals requiring labor-intensive field screening. Thus, GS serves as a decision-support tool that complements conventional breeding, particularly by enhancing the efficiency of progeny selection, as illustrated in Figure 1.

### 3.2. Practical Applicability of GS in Major Crops

Studies consistently demonstrate the practical feasibility of using Genomic Selection (GS) in quantitative disease resistance breeding [51,53,54]. Larkin et al. [51] conducted forward prediction for *Fusarium* head blight (FHB) resistance in soft red winter wheat. They reported that multi-trait GS (MTGS) models, which incorporate auxiliary traits, outperform single-trait GS (STGS) models. In cross-validation, MTGS models achieved average prediction accuracies of 0.66–0.76 across two training environments (TP18_FHB, TP19_FHB), demonstrating performance equivalent to or exceeding traditional phenotypic selection in actual breeding cycles. Notably, in evaluating the accumulation of deoxynivalenol (DON)—a mycotoxin produced by FHB that exhibits high environmental variability—simple phenotypic selection accuracy was only 33.0%, whereas GS accurately selected 69.2–70.1% of superior lines, proving its high potential for practical application. Rolling et al. [54] performed genomic prediction of QDR in soybean germplasm populations. They reported that trait characteristics and marker density more strongly influenced prediction accuracy than the specific statistical model. They found that predictive ability reached near saturation once more than 1000 markers were utilized. Furthermore, the relative efficiency of GS was estimated to be 0.57–0.83 per breeding cycle, suggesting that GS can achieve higher breeding efficiency than phenotypic selection, even for complex disease resistance traits [54]. H. C. Lohithaswa et al. [53] evaluated both QTL mapping and genomic selection in populations resistant to northern corn leaf blight (NCLB). Their findings confirmed that NCLB resistance is a quantitative trait characterized by significant genotype × environment (G × E) interactions and continuous phenotypic distributions. They reported that GS exhibited higher selection efficiency than QTL-based MAS. Additionally, many hybrids derived from high-GEBV lines exhibited intermediate-to-high resistance in field disease evaluations, suggesting that GS can be a practical breeding strategy for complex resistance traits. In another study targeting *Fusarium* stalk rot (FSR) resistance across three doubled haploid populations, prediction accuracy generally improved as training population size and marker density increased. The potential of GS was further supported by a significant positive correlation between resistance in GEBV-based selected lines and their test-cross progeny [55]. Beyond these representative examples, GS has been applied to a wide range of disease resistance traits across major crops. The reported studies include linear models such as RR-BLUP and GBLUP, Bayesian models, kernel-based models, and machine-learning approaches. These applications cover diverse crop–pathogen systems, including *Fusarium* head blight, rusts, powdery mildew, *Phytophthora* resistance, late blight, brown planthopper resistance, and blast resistance. Representative GS models and target disease resistance traits across major crops are summarized in Table 1.

Table 1 shows that GS has been applied using diverse statistical and machine-learning models depending on crop species, disease type, population structure, and trait complexity. Linear models such as RR-BLUP and GBLUP have been widely used because they efficiently capture genome-wide additive effects, whereas Bayesian, kernel-based, and machine-learning models have been explored to account for heterogeneous marker effects, non-linear relationships, and complex genetic architectures. The application of these models across diverse crops, including cereals, legumes, solanaceous crops, and root crops, supports the general utility of GS as a flexible prediction-based strategy for improving quantitative disease resistance. In summary, the accelerated variation in pathogen races driven by climate change is increasingly exposing the limitations of conventional strategies centered on single-gene-dependent qualitative resistance. To address this, quantitative resistance—which offers long-term stable defense with low vulnerability to specific race changes—must become a core breeding objective. GS is a powerful strategy for the early prediction and selection of the total genetic effect of such polygenic resistance traits. However, the predictive power and utility of GS ultimately depend on the breadth and quality of genetic variation within the training population. Therefore, disease resistance breeding in the era of climate change must not only transition to GS-based selection but also move toward restoring and expanding the available genetic diversity.

## 4. Innovations in Diversity Restoration and Genome Decoding: The Pangenome

### 4.1. Genetic Simplification and Vulnerability in Disease Resistance

The next challenge in disease resistance breeding lies beyond applying more sophisticated selection models; it depends on how to secure and interpret the genetic diversity available for practical breeding. Through prolonged selection and improvement focused on traits favorable for productivity, uniformity, and cultivation adaptability, modern cultivars have lost a substantial portion of the genetic diversity required to respond to novel environmental changes and pest pressures [10,71]. A representative example of this is the Irish Potato Famine, which illustrates the inherent vulnerability of relying on genetically uniform cultivars with narrow genetic diversity [71]. Genetic vulnerability due to reduced diversity is commonly observed across various major crops. In wheat, long-term reliance on a limited number of resistance genes, such as *Yr17*, combined with the spread of new stripe rust races like ‘Warrior’ and ‘Kranich’, has exposed the fragility of existing resistance systems [72]. In soybean, repeated reliance on narrow-spectrum resistance genes (e.g., Rps1a, Rps1c, and Rps1k) against *Phytophthora sojae*—a plant-pathogenic oomycete causing root and stem rot—coupled with increased flooding events due to climate change, has accelerated the emergence of new *P. sojae* pathotypes and the instability of traditional resistance [73]. In maize, the combination of a limited genetic background and extreme weather events led to the rapid spread of Tar Spot (*Phyllachora maydis*), resulting in economic losses exceeding $1.37 billion in the United States in 2024 [74]. Furthermore, research on southern corn rust highlights the continuous migration and pathogenic shifts in pathogen populations. Ultimately, genetic simplification must be understood as a structural risk factor that can lead to large-scale disease outbreaks and the collapse of resistance in the era of climate change [75].

### 4.2. Strategies for Gene Pool Expansion and the Integration of Pangenomics

The cases underscore the inherent risks of relying on specific resistance genes or a restricted genetic background. Production systems based on a few elite genotypes or limited resistance gene combinations are likely to become increasingly unstable as climate change intensifies [16,76]. Recent studies suggest that the use of landraces and wild relatives is essential for broadening the genetic base of crop breeding and enhancing resilience to diverse environmental changes [77,78,79]. Future breeding strategies must transition toward actively introducing and recombining diverse germplasm to enhance adaptability to future environmental changes [80]. Excessive reliance on restricted resistance genes has heightened the risk of resistance breakdown and triggered a vicious cycle that narrows the crop’s capacity to respond to new pathogen lineages or environmental shifts [16,76]. To overcome these limitations, efforts have been made to expand the gene pool beyond the utilization of existing elite populations. Multi-parent populations, such as Nested Association Mapping (NAM) and Multi-parent Advanced Generation Inter-Cross (MAGIC), which maximize genetic variation by crossing multiple elite lines, have been actively adopted [81].

These approaches have contributed to increasing the additive genetic standard deviation and broadening the range of useful variations, moving away from conventional methods that relied on single parents [82,83]. Despite these efforts to secure diversity, the shortening of infection cycles due to global warming and the emergence of pathogen races that evolve faster than crop breeding cycles remain significant challenges that are difficult to address through traditional breeding systems alone [23,24,84]. Therefore, it is necessary to integrate various technologies to efficiently utilize expanded populations for quantitative disease resistance breeding. Among these technologies, pangenome-based approaches, which systematically manage species-wide genetic diversity to effectively capture and utilize disease resistance genes, are gaining significant attention. By integrating the genomes of multiple individuals within a species, a pangenome can capture not only the core genome shared by all individuals but also dispensable genes shared by only some and unique genes present in specific individuals [85,86]. This framework enables the effective detection of structural variations (SVs), presence–absence variations (PAVs), and copy number variations (CNVs) that are difficult to identify through single-reference genome analysis. Furthermore, it mitigates the issue of reference bias inherent in single-reference genomes, allowing for a broader capture of previously overlooked genetic differences [87,88].

Recent research on the mango pangenome demonstrates that the choice of reference genome directly impacts the results of population genomics analyses. This study compared conventional reference genomes, haplotype-resolved Telomere-to-Telomere (T2T) genomes, and pangenomes, finding that the pangenome performed best in terms of mapping rates, variant detection, and especially SV detection. While a T2T genome offers high-quality assembly from telomere to telomere, a single genome cannot fully represent the genetic diversity of an entire population. In contrast, the pangenome integrates genomic information from multiple accessions to reduce reference bias and capture variations that might be missing in a single reference. Notably, the fact that Gene Ontology (GO) enrichment results varied depending on the reference genome used indicates that reference bias can affect functional interpretation as well as variant calling. Thus, the pangenome serves as a more comprehensive reference framework that can complement or replace high-quality linear reference genomes [89].

### 4.3. Significance of Pangenomes in the Discovery of Disease Resistance Genes

**Figure 2:**
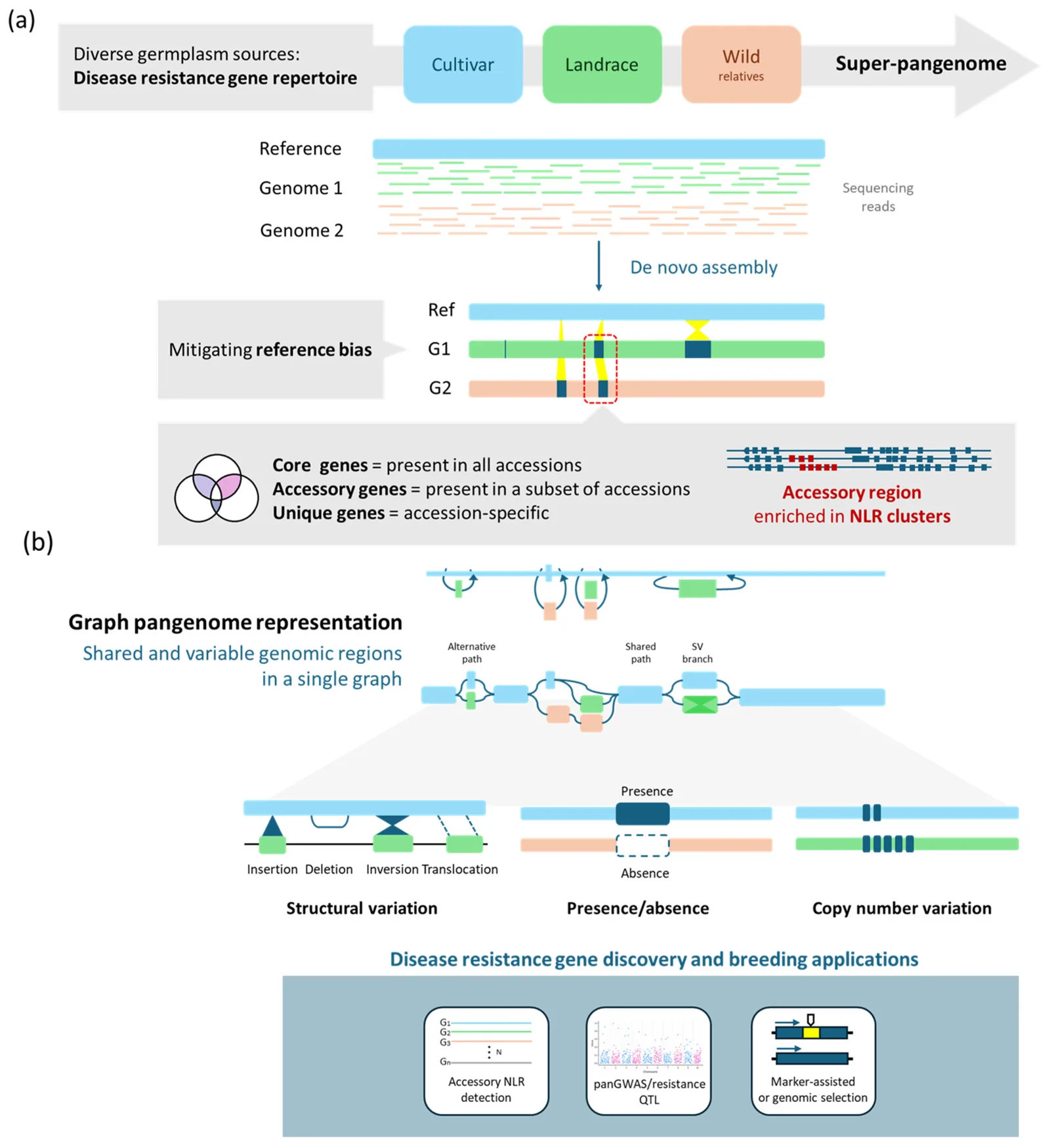
Conceptual framework of the pangenome and structural variation (SV) detection in crops. (**a**) Representation of core, accessory, and unique genomes within diverse germplasms. (**b**) Graph pangenome approach for capturing complex variations including SVs, PAVs, and CNVs.

Recent advancements in genomics have seen a transition from the “map-to-pan” approach, which organizes multiple genomes around a linear reference, to graph-based pangenomes. This method represents the common and branching regions of multiple genomes as a single network (Figure 2) [90,91]. Graph pangenomes have been reported to partially address the “missing heritability” problem often encountered in Genomic Selection (GS) based on genome-wide SNP markers. This is because they can capture structural variations (SVs) that are either strongly associated with or are themselves the causal variants—elements that SNPs alone often fail to detect [91,92]. The value of this pangenome approach is particularly evident in disease resistance research.

The NLR gene family, which plays a pivotal role in pathogen recognition and immune signaling, undergoes rapid evolution and is rich in repetitive sequences. Consequently, these genes exhibit significant differences in Presence–Absence Variation (PAV) and Copy Number Variation (CNV) between individuals [93,94]. Due to these characteristics, disease-resistance-related genes are primarily distributed within the accessory genome, making it difficult to fully reflect their diversity using a single-reference genome [95,96]. Indeed, a study constructing a pangenome based on de novo assemblies of 26 representative soybean lines identified novel sequences not captured by single-reference analysis, including sequences containing NBS-LRR domains associated with various stress responses [97]. Furthermore, a super pangenome study involving 251 accessions of both wild and cultivated Asian and African rice revealed that wild Asian rice harbors a greater number of dispensable NLR genes than cultivated rice. This highlights the existence of resistance gene diversity lost during domestication that remains hidden in single-reference-based analyses [98]. These findings suggest that the pangenome is a highly effective tool for exploring the variable gene families associated with disease resistance. Moreover, pan-GWASs in rice have successfully identified new QTLs and candidate genes for blast resistance that were previously undetectable, proving the utility of the pangenome framework in precisely deciphering the genetic basis of quantitative disease resistance [99].

Recently, there have been active efforts to construct super pangenomes that compare genomes not only within the same species but across the entire genus [100,101,102]. Such advancements are expected to enhance the precise interpretation of genetic diversity, the discovery of trait-associated variants, and the establishment of high-diversity populations for breeding [86,103,104]. Compared to a standard pangenome, a super pangenome provides a broader genus-level analytical framework by integrating cultivars with their wild relatives. This allows for the discovery of lineage-specific genes, rare alleles, structural variations (SVs), and wild-derived genetic resources that are frequently absent in cultivated germplasm [98]. Research on the rice super-pangenome, which utilized Asian cultivated rice (*Oryza sativa*), African cultivated rice (*O. glaberrima*), and their respective wild relatives to construct a graph-based framework, has analyzed variations related to submergence tolerance, seed shattering, and plant architecture. This study compared the independent adaptation and domestication processes of Asian and African rice, confirming that a super-pangenome is instrumental in clearly defining species-specific gene pools [98].

In tomato research, scientists moved beyond the pangenome centered on cultivated varieties and close wild relatives to construct a genus-level super-pangenome encompassing 11 species within the *Solanum* genus. This resulted in the definition of 40,457 pangene families. The study demonstrated that as more genomes were added, the number of gene families increased rapidly, proving that a single-reference genome is insufficient to explain the total genetic diversity of tomatoes. Furthermore, only 54.0% of the total gene families were classified as core, while 38.4% were dispensable and 7.6% were accession-specific. This suggests that a super-pangenome inclusive of wild species is highly effective for discovering lineage-specific genes and wild-derived genetic resources that are difficult to capture in cultivar-centric pangenomes [105]. Pangenomes and super-pangenomes provide a critical framework for reinterpreting the genetic basis of disease resistance by precisely capturing structural variations (SVs), presence–absence variations (PAVs), copy number variations (CNVs), and the diversity of highly variable resistance gene families—all of which are easily missed by single-reference genomes [98,106].

## 5. Multi-Omics Data and AI-Based Precision Breeding

### 5.1. Limitations of Traditional Prediction Models and the Necessity of AI Integration

For the variations captured through pangenomes and the results predicted via GS to exert a practical impact on disease resistance breeding, they must be accompanied by the integration of multi-omics data capable of interpreting the functional consequences of these variations [86,107]. Linear mixed models, such as Genomic Best Linear Unbiased Prediction (GBLUP)—which have been widely used in conventional Genomic Selection (GS)—excel at predicting breeding values from genome-wide markers and estimating additive genetic effects. However, they may face limitations in fully capturing the complex genotype–phenotype relationships and the non-linear, non-additive interactions inherent in high-dimensional multi-omics data [108,109]. To overcome these limitations and interpret the vast “big data” generated in plant breeding, the introduction of non-parametric models based on Deep Learning (DL) and Machine Learning (ML) can be useful [110,111]. Wang et al. [109] demonstrated the potential of deep learning-based models for multi-omics genomic prediction by comparing Deep Neural Network Genomic Prediction (DNNGP) with GBLUP, Light Gradient Boosting Machine (LightGBM), and Support Vector Regression (SVR) [109]. Additionally, Chen et al. [112] demonstrated the complementary utility of non-linear models for sugarcane disease resistance prediction by comparing GBLUP and Bayesian models with an attention-based hybrid model designed to focus on critical SNP markers [112].

### 5.2. Expanding Disease Resistance Interpretation via Multi-Omics and AI Integration

**Figure 3:**
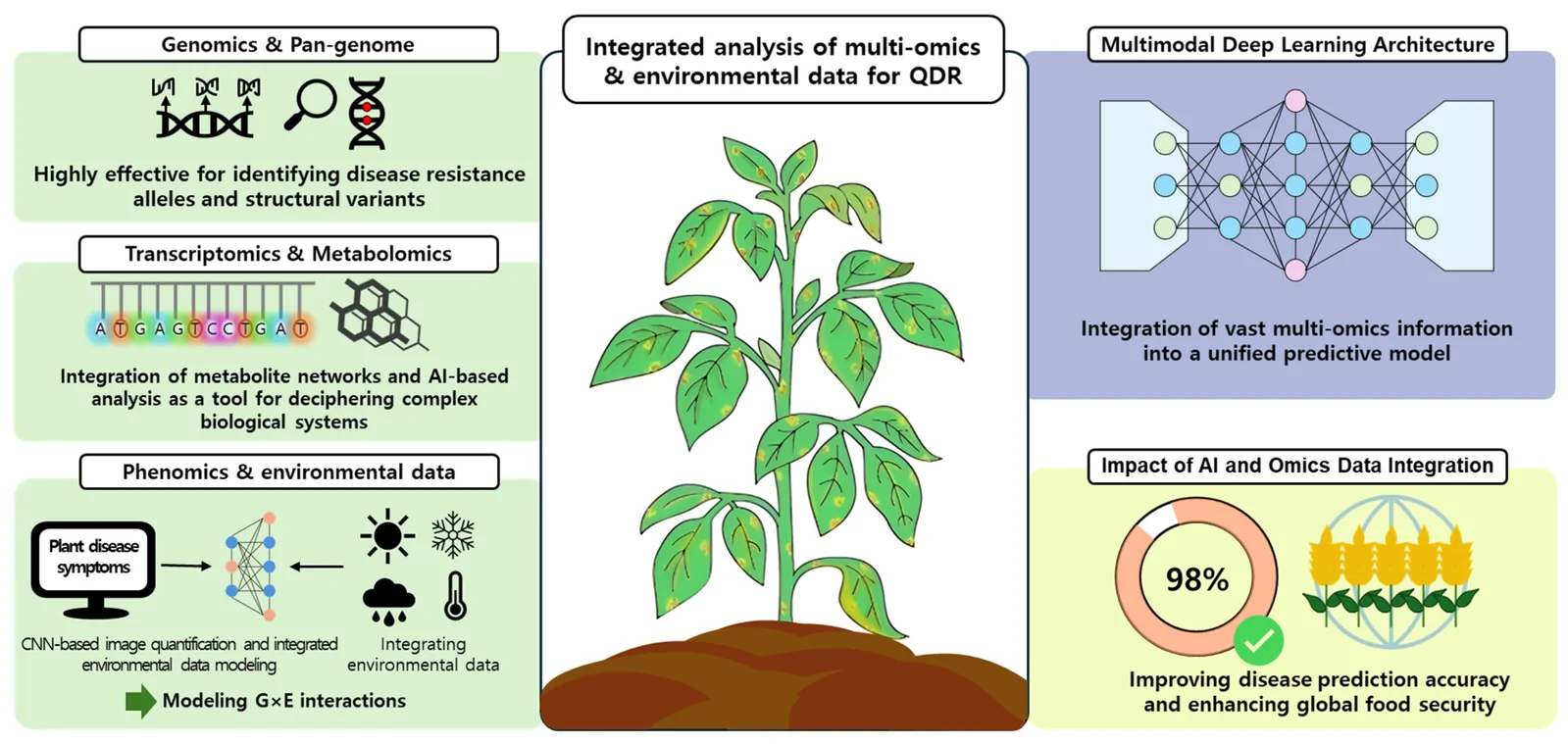
Conceptual framework of AI-driven multi-omics integration for quantitative disease resistance prediction. Genomics, pan-genomics, transcriptomics, metabolomics, phenomics, and environmental data are integrated to characterize disease resistance alleles, molecular responses, symptom patterns, and genotype-by-environment (G × E) interactions. Multimodal deep learning models can combine these heterogeneous data layers into a unified predictive framework, thereby improving disease prediction accuracy and supporting precision breeding for quantitative disease resistance.

AI and machine-learning methods can address these challenges by identifying nonlinear relationships and integrating heterogeneous data layers into unified predictive frameworks [113,114]. Within this integrated framework (Figure 3), genomics and pangenomics provide the foundational genetic layer for identifying the genome-wide variation underlying disease resistance.

- Genomics & Pangenomics

Conventional single-reference genomes may fail to capture complex variations frequently observed in disease resistance regions, such as structural variations (SVs), presence/absence variations (PAVs), and copy number variations (CNVs) [115,116]. These findings highlight the utility of pangenomes in discovering disease-resistance-related genetic variants that were overlooked in traditional single-reference-based analyses. Furthermore, integrating pangenome-derived SV/PAV information with GWAS and interpretable machine learning enables the prioritization of candidate variants associated with complex traits. This facilitates an accelerated search for superior disease resistance alleles existing in wild relatives and non-elite germplasm [117].

- Transcriptomics & Metabolomics

In this context, AI refers to supervised machine-learning models that use matched gene-expression and metabolite-abundance profiles as predictors. Random forests, regularized regression, or neural networks can model nonlinear relationships and prioritize genes or metabolites associated with disease response phenotypes. Feature-importance or attribution analyses can identify candidates for experimental testing, but predictive associations alone do not establish that a gene controls metabolite production or secretion [113,114]. Conventional omics integration provides the biological context for these analyses [118,119,120].

Toubiana et al. [121] constructed correlation networks between metabolites in tomato and used machine learning to predict metabolic pathways with a high probability of existence. Some of these predicted candidates were further validated through gene expression analysis, enzymatic activity assays, and metabolite detection. This case demonstrates that combining metabolite networks with AI-based analysis can uncover novel metabolic pathway candidates, highlighting the potential of AI-driven multi-omics interpretation [121].

- Phenomics & Environmental Data

For phenomic and environmental data, CNNs can convert RGB or hyperspectral crop images into disease class probabilities, lesion estimates, or spectral features. These image-derived phenotypes can then be combined with genome-wide markers and measured environmental covariates, such as temperature, rainfall, humidity, and soil properties, in kernel-based, tree-based, or multimodal neural network models. The intended breeding outputs are predicted disease severity or resistance performance of candidate lines in specified environments, rather than image labels alone. Training and validation should keep years, locations, or genotypes separate where appropriate to assess transfer to unseen breeding conditions and avoid information leakage. Multi-environment genomic prediction and image-based phenotyping support components of this proposed workflow; an integrated model still requires crop-specific validation [122,123,124,125,126,127].

Kuswidiyanto et al. [125] reviewed the applicability of aerial hyperspectral imaging and deep learning-based classification models for plant disease diagnosis, suggesting that these image-based approaches can serve as phenomic tools for rapid, quantitative symptom detection. Notably, deep learning-based symptom recognition studies have demonstrated that disease symptoms can be classified with high accuracy from plant images, with reported diagnostic accuracies reaching approximately 98% [125]. This approach plays a critical role in the data-driven precision breeding strategy proposed in this review for interpreting complex genotype-by-environment (G × E) interactions. Disease resistance is not determined solely by the presence of specific resistance genes but is shaped by the interaction among genotypes, pathogens, growth stages, and various environmental factors such as temperature, humidity, and light conditions [128]. Therefore, image-based deep learning functions as a high-speed, objective phenotyping layer. When integrated with genomics, transcriptomics, metabolomics, and environmental data, it enables a more precise prediction of complex phenotypic variation in quantitative disease resistance (QDR). Image-based disease detection demonstrates one component of this framework [129], whereas linking image features with genomic and molecular data for QDR prediction remains a proposed extension that requires matched datasets and independent validation. Thus, deep learning based on symptom imagery should be interpreted not merely as a disease classification technology, but as a core phenomics component designed to quantify phenotypes in QDR breeding and link genomic, omic, and environmental data into a single predictive model [130]. Such integrated analyses contribute to modeling disease resistance responses that vary according to environmental conditions, improving disease prediction accuracy and fostering the development of precision selection strategies. For breeding decisions, the model outputs can be used to rank candidate progenies for field testing and to prioritize crossing combinations. Their value should be judged against established genomic prediction models and subsequent phenotypic performance, rather than assumed from model complexity alone [108,131,132].

## 6. Conclusions

Rapid climate change and complex environmental stressors have altered pathogen life cycles and accelerated the emergence of new races, placing traditional qualitative resistance and MAS-centered breeding—which rely on a limited number of major genes—at a critical risk of failure. To address these challenges, breeding strategies should place greater emphasis on quantitative disease resistance (QDR), which can contribute to durable resistance but does not inherently guarantee stability across changing environments. Its effectiveness should therefore be evaluated across relevant pathogen isolates and environments. GS can support this objective by predicting genomic estimated breeding values for quantitative resistance, provided that the training populations and validation designs represent the target pathogen pressures and environments. Furthermore, to restore the genetic diversity lost in modern cultivars, a pangenome approach must be adopted to broadly identify structural variations (SVs) and complex disease resistance gene families (such as NLRs) that are often missed by single-reference genomes. To decipher the biological mechanisms of these discovered variants, an AI-based multi-omics precision breeding strategy—integrating genomic, transcriptomic, metabolomic, phenomic, and environmental data—must be implemented. However, for pangenome-based diversity expansion and AI-driven G × E (genotype-by-environment) interaction prediction models to yield practical results in the field, the systematic construction and management of thoroughly standardized data are fundamental prerequisites. Data for precision breeding should be standardized into a three-layer structure:

1. Observation Layer: Containing genotypes and multi-layered omics data.
2. Contextual Information Layer: Including evaluation of environments and disease pressure indicators.
3. Quality and Traceability Layer: Ensuring reproducibility and accurate individual matching.

Multi-omics information combined with comprehensive environmental and cultivation metadata goes beyond simple DNA variations to clearly explain the complex biological pathways through which traits are expressed, thereby drastically enhancing the generalization performance of AI prediction models.

In conclusion, future breeding to ensure food security in the era of climate change will be realized through the organic integration of gene pool expansion via pangenomes, non-linear trait prediction using GS and AI, and a three-layer standardized data integration system. Future breeding research must focus on strictly standardizing trait definitions and metadata formats across multiple years and institutions. This data-driven decision-making framework will serve as the core foundation for selecting stable resistant lines and proactively designing crossing combinations amidst weather anomalies and rapid pathogen evolution.

## Figures and Tables

**Table 1:** Summary of Genomic Selection (GS) models for disease resistance across major crops. This table summarizes representative genomic selection and genome-wide prediction studies for disease resistance traits in major crops. The listed models include linear, Bayesian, kernel-based, and machine-learning approaches, covering diverse traits such as FHB, rust, powdery mildew, *Phytophthora* resistance, late blight, brown planthopper resistance, and blast resistance.

Models	Crops	Traits	References
RR-BLUP, LASSO, Elastic net	Wheat	FHB-related traits	[56]
RR-BLUP, BayesA, BayesB, BayesC, BLASSO, BRR, RKHS	Wheat	FHB-related traits: INC, SEV, FDK, DON	[57]
RR-BLUP	Wheat	FHB resistance/severity	[58]
RR-BLUP	Wheat	*Septoria tritici* blotch resistance	[59]
GBLUP	Wheat	Barley yellow dwarf resistance/severity	[60]
GBLUP	Barley	Powdery mildew, leaf rust, ryegrass yellow virus resistance	[61]
Decision Tree, Random Forest, Neural Network, Gaussian Process Regression	Barley	Powdery mildew resistance	[62]
RR-BLUP, Bayesian power lasso	Wheat/barley	Yield, quality, disease-related traits	[63]
Bayesian Ridge Regression, Bayesian LASSO, BayesA, BayesB, BayesC, RKHS	Soybean	*Phytophthora sojae* resistance	[54]
Bayesian Lasso	Soybean	Quantitative disease resistance to *Phytophthora sojae*	[64]
RR-BLUP, GBLUP, Bayesian LASSO, Random Forest, SVM	Soybean	*Phakopsora pachyrhizi* resistance	[65]
RR-BLUP	Maize	Common rust (*Puccinia sorghi*)	[66]
RR-BLUP	Maize	Maize lethal necrosis disease resistance	[67]
GBLUP	Potato	Late blight, potato cyst nematode resistance	[68]
RR-BLUP, GBLUP, Bayesian LASSO, BSLMM, Random Forest, ANN, SVM, KAML	Rice	Brown planthopper resistance	[69]
Single-trait GBLUP, multi-trait GBLUP, weighted GBLUP	Rice	Blast resistance	[70]

## Data Availability

No new data were created or analyzed in this study. Data sharing is not applicable to this article.
